# Recent range-wide demographic expansion in a Taiwan endemic montane bird, Steere's Liocichla (*Liocichla steerii*)

**DOI:** 10.1186/1471-2148-10-71

**Published:** 2010-03-10

**Authors:** Bailey D McKay, Herman L Mays, Yi-Wen Peng, Kenneth H Kozak, Cheng-Te Yao, Hsiao-Wei Yuan

**Affiliations:** 1 Bell Museum of Natural History, University of Minnesota, St Paul, MN 55108, USA; 2 Department of Ecology, Evolution and Behavior, University of Minnesota, St Paul, MN 55108, USA; 3 Geier Collections and Research Building, Cincinnati Museum Center, Cincinnati, OH 45203, USA; 4 School of Forestry and Resource Conservation, National Taiwan University, Taipei, Taiwan; 5 Department of Fisheries, Wildlife & Conservation Biology, University of Minnesota, St Paul, MN 55108, USA; 6 Endemic Species Research Institute, Council of Agriculture, Jiji, Taiwan; 7 Department of Life Sciences, National Cheng Kung University, Tainan, Taiwan

## Abstract

**Background:**

The subtropical island of Taiwan is an area of high endemism and a complex topographic environment. Phylogeographic studies indicate that vicariance caused by Taiwan's mountains has subdivided many taxa into genetic phylogroups. We used mitochondrial DNA sequences and nuclear microsatellites to test whether the evolutionary history of an endemic montane bird, Steere's Liocichla (*Liocichla steerii*), fit the general vicariant paradigm for a montane organism.

**Results:**

We found that while mountains appear to channel gene flow they are not a significant barrier for Steere's Liocichla. Recent demographic expansion was evident, and genetic diversity was relatively high across the island, suggesting expansion from multiple areas rather than a few isolated refugia. Ecological niche modeling corroborated the molecular results and suggested that populations of Steere's Liocichla are connected by climatically suitable habitat and that there was less suitable habitat during the Last Glacial Maximum.

**Conclusions:**

Genetic and ecological niche modeling data corroborate a single history--Steere's Liocichla was at lower density during the Last Glacial Maximum and has subsequently expanded in population density. We suggest that such a range-wide density expansion might be an overlooked cause for the genetic patterns of demographic expansion that are regularly reported. We find significant differences among some populations in *F*_*ST *_indices and an admixture analysis. Though both of these results are often used to suggest conservation action, we affirm that statistically significant results are not necessarily biologically meaningful and we urge caution when interpreting highly polymorphic data such as microsatellites.

## Background

Past climatic cycles have had a profound impact on the levels and distribution of genetic diversity [1]. Despite the fact that most biodiversity occurs in tropical areas, most studies have focused on the temperate regions in North America and Europe where the retreat of ice sheets has left widespread genetic patterns consistent with northward expansion from southern refugia [2]. This bias has left us with a poorer understanding of the impact of climate cycles on the phylogeographical patterns of tropical taxa. However, initial phylogeographic studies indicate that tropical areas have been impacted by climatic cycles and that tropical species can have deep intraspecific lineages with complex histories [2,3].

**Figure 1:**
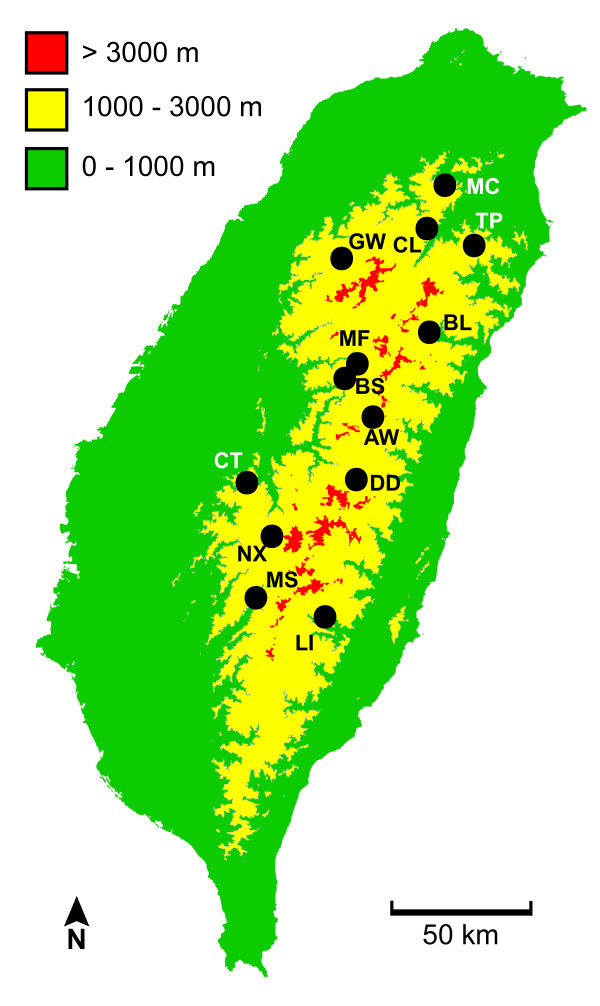
**Map of Taiwan showing elevation and sampling site locations**. Colors denote elevation. Sampling sites are indicated by black circles. The dotted line represents a phylogeographic division reported for many taxa. The yellow area (between 1000 m and 3000 m is the approximate range of Steere's Liocichla). Population abbreviations are from Table 1.

The subtropical island of Taiwan is an area of high endemism as well as topologic, ecologic, and climatic diversity. Taiwan first emerged from the sea following the collision of the Eurasian and Philippine Sea plates approximately 5 mya [4,5]. Despite periodic connections with the mainland during low sea levels [6], Taiwan's general isolation from mainland Asia has given rise to many endemic species and subspecies. Within the island, mountains have provided additional opportunities for isolation. This is reflected in the phylogeographic patterns found in some lowland taxa. For example, a freshwater crab (*Candidiopotamon rathbunae*) [7], a frog (*Fejervarya limnocharis*) [8], and a gecko (*Gekko hokouensis*) [9] exhibit a common pattern of genetic division east and west of the Central Mountain Range (CMR), the prominent mountain range that runs through much of the longitudinal length of the island (Fig. 1). In contrast, many montane species show a pattern of north-south genetic division: e.g. Formosan Wood Mouse (*Apodemus semotus*) [10], a ranid frog (*Rana sauteri*) [11], and Siberian Weasel (*Mustela sibirica*) [12]. The observation of common phylogeographic patterns in such varied taxa suggests vicariance as the cause [13].

The east-west pattern of genetic division has been found in lowland birds (i.e. Light-vented and Taiwan Bulbul *Pycnonotus sinensis *and *P. taivanus*, [14]; Ring-necked Pheasant *Phasianus colchicus*, [15], however, no montane bird has yet been phylogeographically surveyed. Determining whether montane birds were affected by the same isolating events that generated genetic divisions in other montane taxa is important for understanding the impact of mountain vicariance in Taiwan. In a conservation context, uncovering historical lineages is essential for guiding management efforts, especially for species confined to single islands. Steere's Liocichla (*Liocichla steerii*) is a montane, resident bird species endemic to Taiwan. It inhabits forest edge and secondary growth from approximately 1000 m to 3000 m. Its dispersal ability was inferred to be low [16], and high song diversity also suggests dispersal may be low [17]. Therefore, Steere's Liocichla is an ideal avian candidate to have developed the same genetic structure observed in potentially less vagile vertebrates.

In this paper, we used mitochondrial DNA sequences and nuclear microsatellites to investigate the evolutionary history of Steere's Liocichla. Our primary goal was to determine whether this endemic species showed the same phylogeographic patterns of deep divergence that characterize other montane vertebrates in Taiwan. As a supplement to our genetic data, we used ecological niche modeling to predict this species' range during the Last Glacial Maximum (LGM). We also considered whether any populations of Steere's Liocichla might be of special management concern for conservation efforts.

## Methods

### Sampling and laboratory methods

A total of 122 individuals was sampled from 13 populations located throughout the breeding range of the species (Fig. 1; Table 1). Birds were caught during the breeding season (March to September) by mist net and 50 *μ*l of blood was collected via brachial venipuncture. Blood samples were stored in lysis buffer [18] at room temperature until they could be transported to the laboratory where they were stored at -20°C.

**Table 1:** Intrapopulation statistics for each Steere's Liocichla population separately and for all samples combined

			mtDNA					microsatellites					
				
Population			n	π	nh	h	F_S_	n	A	A_R_	H_O_	H_E_	F_IS_
Aowanda	AW	South	3	0.00260	3	1.00	-0.34	4	4.00	4.29	0.86	0.73	-0.171
Beidongyanshan	BS	North	9	0.00184	4	0.69	0.27	11	5.43	4.00	0.80	0.72	-0.121
Bilu	BL	North	7	0.00446	7	1.00	-3.23*	11	6.14	4.42	0.70	0.80	0.119
Chilan	CL	North	3	0.00195	3	1.00	-0.69	5	4.00	4.10	0.80	0.75	-0.062
Chitou	CT	South	9	0.00309	5	0.81	0.17	9	5.43	4.48	0.74	0.80	0.073
Danda	DD	South	9	0.00309	6	0.92	-1.03	12	6.29	4.26	0.72	0.77	0.060
Guanwu	GW	North	9	0.00271	6	0.89	-1.35	11	6.00	4.48	0.67	0.79	0.149
Liyuan	LI	South	10	0.00143	5	0.76	-1.32	13	7.57	4.81	0.84	0.82	-0.034
Mingchi	MC	North	7	0.00912	6	0.95	-2.20*	10	5.43	4.47	0.85	0.80	-0.070
Meifeng	MF	North	10	0.00236	7	0.87	-2.67*	10	6.71	4.80	0.81	0.83	0.015
Moshan	MS	South	8	0.00164	5	0.79	-1.54	9	5.43	4.42	0.75	0.79	0.050
Nanxi	NX	South	1	n/a	n/a	n/a	n/a	7	5.14	4.39	0.60	0.80	0.245
Taipingshan	TP	North	6	0.00286	5	0.93	-1.33	10	6.57	4.65	0.74	0.81	0.087
Total			91	0.00272	36	0.90	-26.6**	122	5.70	4.43	0.76	0.79	0.026

The summary of genetic diversity includes sample size (n) nucleotide diversity (π), number of haplotypes (nh), haplotype diversity (h), Fu's F_S _(F_S_), average number of alleles per locus (A), allelic richness (A_R_), observed heterozygosity (H_O_), expected heterozygosity (H_E_), and inbreeding coefficient (F_IS_). Microsatellite statistics are based on the average of seven polymorphic loci.

* P < 0.05; ** P < 0.001

Whole genomic DNA was extracted using a standard phenol-chloroform protocol followed by ethanol precipitation. The mitochondrial NADH dehydrogenase subunit 2 (ND2) gene was amplified from a subset of 91 individuals representing all populations (Table 1) using the primers L5216 and H6313 [19]. Polymerase chain reaction (PCR) was performed in 10 *μ*l reactions on a MJ Research PTC-100 thermocycler using a thermal profile of 94°C for 4 min followed by 30 cycles of 1 min at 94°C, 1 min at 55°C, and 2 min at 72°C, and then 10 min at 72°C. Primers and excess dNTPs were removed from the PCR product with ExoSAP-IT (USB Corporation) following the manufacturer's instructions. Sequencing was performed on an ABI 3700 automated sequencer using BigDye kit v. 3.0 according to recommended protocols (Applied Biosystems). All products were sequenced in both directions. Usually, the amplification primers were sufficient to obtain >1,000 base pairs (bp) of unambiguous sequence. However, whenever an ambiguous stretch of sequence was produced, internal sequencing primers (L5758 and H5766; [19]) were used to clarify ambiguous bases. Complementary strands were aligned and edited using SEQUENCHER v. 4.6 (GeneCodes Corporation). All sequences were inspected individually using the raw spectrograph data and every point mutation was checked for authenticity. Sequences have been deposited in GenBank (GU560065-GU560155).

All individuals were also genotyped at seven microsatellite loci. Four loci were developed specifically for Steere's Liocichla (lsgata17, lsgata 21, lsgata 24, lsgata 25; [20]) and three additional loci (GC-GATA10, GC-GATA 15, GC-GATA 23; [21]) were originally developed for another babbler, the Hwamei (*Garrulax canorus*). Forward primers were fluorescently labeled, and the PCR cycling protocol followed the above for all loci except GC-GATA 15, for which we used an annealing temperature of 50°C. Fragment analysis of the amplification product was conducted on a Beckman CEQ 8000 sequencer (Beckman Coulter).

### Genetic data analysis

For mtDNA sequences, we estimated nucleotide diversity (π), number of haplotypes (nh), and haplotype diversity (h) for each population using the program DnaSP v. 4.9 [22]. For each population, we also computed Fu's F_S_, a neutrality statistic that is particularly sensitive to population demographic expansion [23]. Its significance was tested with 10,000 coalescent simulations using the program ARLEQUIN 3.11 [24]. We used a mismatch distribution analysis to estimate parameters of a demographic expansion using a generalized least-square approach [25], as implemented in ARLEQUIN. To estimate the time of expansion, we used the formula τ = 2 *μt *[26], where τ is the time since the demographic expansion, *t *is the number of generations, and *μ *is the mutation rate. We obtained a 95% confidence interval (CI) for τ with 1000 replicates of bootstrapping in ARLEQUIN. We tested overall genetic structure of populations with a hierarchical analysis of molecular variance (AMOVA; [27]) in ARLEQUIN. To test for restricted gene flow between the north and south groups identified by previous studies, we assigned populations to either a "north" or "south" group. Based on the study of Hsu et al. [10], we used the Choushui River as the boundary between groups (see Table 1). To visualize haplotype relationships, we constructed a haplotype network using the parsimony-based algorithm of Templeton et al. [28] and implemented in the program TCS 1.21 [29]. Networks are generally better than bifurcating trees at representing the relationships of intraspecific phylogenies [30].

Microsatellite genotypes were tested for departures from Hardy-Weinberg equilibrium and evidence for linkage disequilibrium using ARLEQUIN. To assess allelic variation within populations, we calculated allele frequencies, observed (Ho) and expected (He) heterozygosities using GENEPOP 4.0.9 [31]. To correct for sample size differences, we calculated allelic richness using rarefaction with the program FSTAT 2.9.3 [32,33]. A sequential Bonferroni correction was applied to correct for multiple comparisons [34]. Principal component analysis (PCA) was applied to allele frequency data as a measure of strong multilocus associations among populations. We performed a PCA with arcsinsquare root transformed allelic frequencies using SAS (SAS Institute).

To assess genetic structure between populations, we computed pairwise *F*_*ST *_and *R*_*ST *_values for all populations. *R*_*ST *_is analogous to *F*_*ST *_but incorporates a stepwise mutation model of evolution that was specifically designed for microsatellite data [35]. Next, we performed another hierarchical AMOVA to determine the proportions of microsatellite variation among and within populations. Groups were defined in the same way as for the mtDNA AMOVA. Fixation indices and AMOVA were computed in ARLEQUIN. We also inferred genetic population structure using a Bayesian assignment approach implemented in the program BAPS 5.2 [36,37]. BAPS has been shown to perform better than STRUCTURE when F_ST _is small [38]. We began with a mixture analysis to determine the most probable number of populations (*K*) given the data. We performed the "clustering of groups of individuals" analysis, considering clusters to contain a minimum of three individuals. We estimated the number and composition of clusters considering upper limits of *K *at 2-10, 15, and 20. We ran 10 repetitions for each maximum *K*. Using the groups identified in the mixture analysis, we conducted an admixture analysis with 100 realizations from the posterior of the allele frequencies. We repeated the admixture five times to confirm consistent results. We also performed a "spatial clustering of groups" analysis that incorporates geographical information in the estimation of the genetic population structure [37]. This approach provides more statistical power to infer structure when molecular data are weak. The spatial mixture analysis followed the above procedure.

Finally, to test for an association between genetic distance (*R*_*ST*_) and geographic distance, we performed a Mantel test using GenAlEx 6 [39]. We ran two Mantel tests with different geographic distances. The first used "straight-line" distances, which were simply the shortest distances between populations. However, Steere's Liocichla has a restricted altitudinal range and is therefore unlikely to traverse very high or very low altitudes. Thus, the second Mantel test used "least-cost" distances, which were calculated as the shortest distances between populations assuming travel was limited to elevations between 1000 m and 3000 m. Straight-line and least-cost geographic distances were calculated in ArcGIS 9 (ESRI).

### Ecological niche modeling

We used ecological niche modeling (ENM) to evaluate our molecular analyses of demographic history and population structure. Briefly, the general ENM approach applied here generates a map of the expected distribution of a species using data on the environmental conditions where it is known to occur and randomly selected background locations in the study area [40]. We used Maxent version 3.2 [41] to model the potential geographic distribution of Steere's Liocichla. Maxent is a general approach for characterizing probability distributions from incomplete information [41]. Maxent computes a probability distribution that describes the relative suitability of each grid cell as a function of the environmental variables at all the known occurrence locations, and when projected into geographic space it produces a map of the species' potential geographic distribution.

To construct the model, we used 19 bioclimatic variables from the WorldClim dataset with 2.5-minute spatial resolution [42] and 286 georeferenced breeding-season localities for Steere's Liocichla. The climatic variables are derived from weather station data and quantify annual variation in temperature and recipitation. Occurrence records within the same map pixel were removed to avoid pseodoreplication. To calibrate the model, we used quadratic features, and default parameters for the number of background pixels, regularization, the convergence threshold, and the maximum number of iterations. We randomly selected 75% of the occurrence locations to construct the model; the remaining 25% were set aside to test the model. We calculated the area under the receiving operator characteristic (AUC) to test whether the model could discriminate between the test localities and 10,000 localities randomly selected from across Taiwan. Finally, we selected logistic output, which ranges from 0-1 and quantify the probability of suitable environmental conditions for the species in each grid cell.

To estimate the geographic distribution and extent of suitable climate for Steere's Liocichla at the LGM (21,000 years before present), we applied the contemporary ecological niche model to a set 19 bioclimatic variables generated from CCSM3 paleoclimatic model. In doing so, we used the fade-by-clamp option in Maxent version 3.2 to remove heavily clamped pixels from the final prediction. Failing to remove such pixels from the region might lead to erroneously extensive predictions of suitable climate when transferring a model to a different time period.

Distribution-based niche modeling makes at least three important biological assumptions. First, it assumes that a species distribution is at equilibrium with the present-day climate conditions. Second, it assumes that the distribution of the species is not strongly influence by biotic factors (e.g. competitors or predators). Third, it assumes that the climatic tolerances of the species are conserved over the timescale for which its distribution is being modeled. If any of these conditions is violated, the extent of climatically suitable habitat could be underestimated.

## Results

### mtDNA

**Figure 2:**
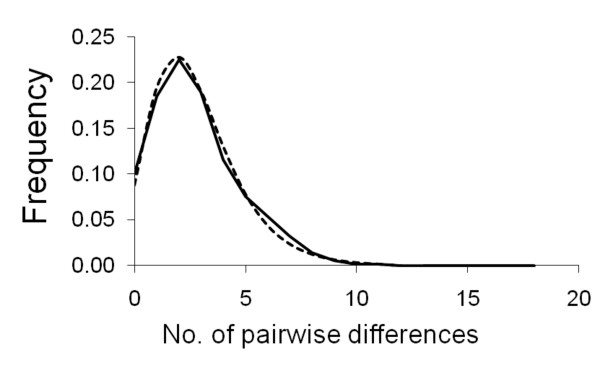
**Mismatch distribution of Steere's Liocichla mitochondrial sequence data**. There is a significant correlation between observed (solid line) and expected frequencies under a model of sudden expansion (dotted line) for the number of pairwise differences.

**Figure 3:**
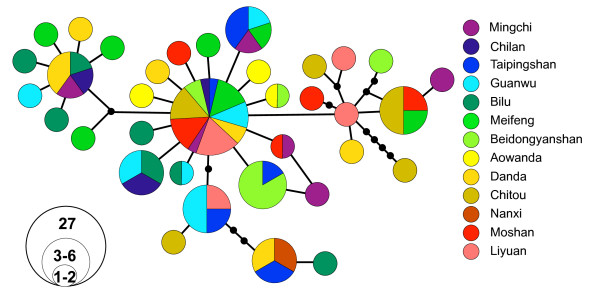
**Mitochondrial DNA haplotype network for Steere's Liocichla ND2 sequence data**. Colors indicate the population of origin. Populations from the northern group are represented by cool colors (blues and greens), and populations from the southern group are represented by warm colors (reds and yellows). Each circle represents a haplotype, and the size of the circles is proportional to its frequency. Small black circles represent unsampled haplotypes.

A total of 1026 bp was obtained from the mitochondrial ND2 gene. There were 48 polymorphic and 20 parsimony informative sites resulting in 36 unique haplotypes. Nuclear copies of mitochondrial genes are known in birds [43]. However, the large number of observed haplotypes and an absence of nonsensical stop-codons support a mitochondrial origin for our sequences [44]. Overall nucleotide diversity (π) was 0.00272 for all samples combined and ranged from 0.00143 to 0.00446 (Table 1). Overall haplotype diversity was 0.9 and ranged from 0.69 to 1.00 (Table 1). AMOVA indicated non-significant differentiation (1%) between north and south groups and significant population differentiation, with 4.5% (P < 0.001) of the molecular variance partitioned among populations (Table 2). The mtDNA mismatch distribution was distinctly unimodel and did not differ significantly from a model of sudden expansion (Fig. 2). The estimate of τ was 2.9 (95% CI 0.7-5.5). Using an avian ND2 mutation rate of 5.4 × 10^-8 ^[45] and assuming a generation time of two years, the time of expansion was estimated to have occurred approximately 26000 (95% CI 6300-49700) ybp. The haplotype network showed no obvious genetic structure (Fig. 3).

**Table 2:** Analyses of molecular variance (AMOVAs) showing the distribution of genetic variation among populations of Steere's Liocichla on Taiwan

Source of variation	mtDNA				microsatellites			
		
	df	% total	*F*-statistic	*P*-value	df	% total	*F*-statistic	*P*-value
Among groups (north v. south)	1	0.98	0.010	0.140	1	-0.20	-0.002	0.713
Among populations Within groups	11	4.48	0.045	0.011	11	1.92	0.019	< 0.001
Within populations	78	94.54	0.055	0.003	231	98.28	0.017	< 0.001

### Microsatellites

The microsatellite dataset showed no statistically significant deviations from Hardy-Weinberg equilibrium or evidence of lineage disequilibrium. There were a total of 137 alleles, and the average number of alleles per loci was 19.6. The number of alleles per locus ranged from 9 in lsgata21 to 51 in lsgata17. Allelic richness ranged from 4.00 in Aowanda to 7.57 in Liyuan (Table 1). PCA resulted in relatively little explanatory power with only 15.6% and 12.6% of the variation explained by the first and second principal components, respectively. It took seven principal components to explain 75% of the variation. This suggests weak multilocus associations among populations.

Overall, our analyses of population structure yielded conflicting results with different populations highlighted by different analyses. *F*_*ST *_pairwise population comparisons resulted in significant differences between the Beidongyanshan population and every other population (Table 3). *R*_*ST *_pairwise indices were larger that *F*_*ST *_comparisons (Table 3). This is expected because *F*_*ST *_estimated from highly variable markers is known to bias estimates downwards. This is because the proportion of the total variation among populations can never be very high when there is a lot of variation within populations [46]. *R*_*ST *_comparisons did not highlight the Beidongyanshan population as particularly distinct. Rather, there were several significant comparisons that seem haphazard with respect to geography (Table 3). AMOVA of microsatellite alleles indicated nonsignificant amounts of differentiation (0%) distributed between north and south groups and little but significant population differentiation with 1.9% (P < 0.001) of the variance distributed among populations (Table 2).

**Table 3:** Pairwise estimates of *F*_*ST *_(below diagonal) and *R*_*ST *_(above diagonal) among 13 populations of Steere's Liocichla

	AW	BS	BL	CL	CT	DD	GW	LI	MC	MF	MS	NX	TP
AW		0.063	0.000	0.028	**0.325**	0.018	0.135	**0.363**	0.000	0.251	0.175	0.210	0.081
BS	**0.062**		0.039	0.000	**0.202**	0.000	0.047	**0.265**	0.045	**0.246**	0.050	0.129	0.000
BL	0.029	**0.044**		0.041	**0.288**	0.055	0.124	**0.370**	0.000	**0.301**	**0.194**	**0.219**	0.076
CL	0.051	**0.097**	0.013	3	0.057	0.000	0.000	0.157	0.000	0.173	0.018	0.024	0.000
CT	0.050	**0.043**	0.016	0.004		0.063	0.000	0.000	**0.222**	0.181	0.005	0.000	0.034
DD	0.000	**0.036**	0.006	0.018	0.027		0.000	0.159	0.000	**0.240**	0.042	0.010	0.000
GW	0.000	**0.049**	0.017	0.036	0.000	0.008		0.096	0.049	**0.213**	0.012	0.000	0.000
LI	0.035	**0.049**	0.019	0.018	0.000	0.006	0.001		**0.280**	**0.194**	0.053	0.018	0.121
MC	0.031	**0.043**	0.019	0.001	0.015	0.000	0.025	0.020		**0.267**	0.076	0.121	0.021
MF	0.050	**0.055**	0.000	0.008	0.006	0.012	0.019	0.004	0.020		0.193	0.000	0.184
MS	0.035	**0.046**	0.009	0.020	0.013	0.020	0.015	0.011	0.000	0.017		0.000	0.016
NX	0.073	**0.131**	0.003	0.000	0.006	0.027	0.024	0.013	0.000	0.002	0.000		0.000
TP	0.009	**0.039**	0.002	0.027	0.000	0.000	0.000	0.008	0.000	0.002	0.000	0.023	

Bolding indicates a significant statistic.

**Figure 4:**
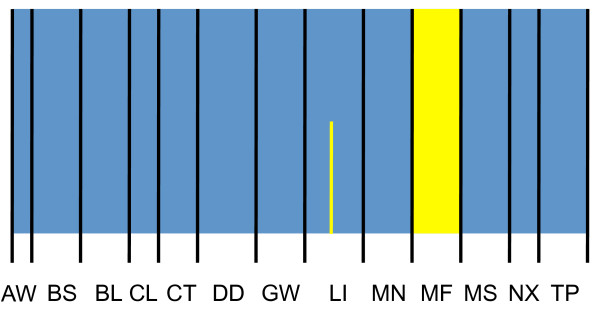
**Admixture coefficients for Steere's Liocichla estimated using BAPS**. Vertical columns correspond to individuals; black lines separate populations. Two groups were identified and they are indicated by different colors. Columns are colored in proportion to the estimated admixture coefficients for each individual. Population abbreviations are from Table 1.

The mixture analysis in BAPS indicated the presence of two distinct groups of populations, with the Meifeng population the sole member of one group and all other populations comprising the other group. The admixture analysis assigned almost every individual to the correct group (Fig. 4). In contrast, the spatial analysis that incorporated geographic information about populations suggested only a single group. The Mantel test indicated a positive but non-significant correlation between genetic (*R*_*ST*_) and geographic distances (km) when straight-line geographic distances were used (R^2 ^= 0.01; P = 0.23). However, when least-cost geographic distances were considered, the Mantel test resulted in a positive and significant correlation (R^2 ^= 0.07; P = 0.04). This suggests that dispersal for these birds is, at least somewhat, limited by altitude.

### Ecological niche modeling

**Figure 5:**
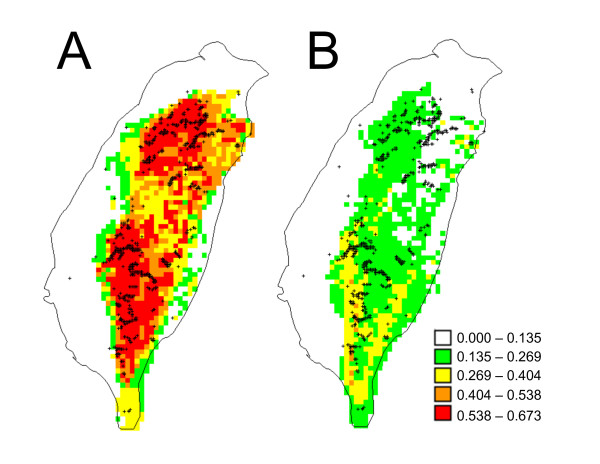
**Predicted geographic distribution of Steere's Liocichla based on (a) present-day climatic conditions and (b) climatic conditions at the Last Glacial Maximum**. Grid cells are classified by predicted suitability with white being least suitable and red being most suitable. Georeferenced occurrence records used to generate the models are indicated by plus signs.

The predicted geographic distribution of Steere's Liocichla for the present-day and LGM are shown in Fig. 5. The area under the receiving operator characteristic demonstrates that the model discriminated strongly between randomly selected locations across Taiwan, and the training (AUC = 0.93) and test localities (AUC = 0.97).

Intriguingly, despite the many assumptions that are made when constructing distributionbased niche models, both the contemporary and LGM predictions are consistent with the findings of the molecular analyses. The model predicts that populations of Steere's Liocichla are connected by climatically suitable habitats (Fig. 5A). This result corroborates the analyses of population structure, which suggest that the degree of gene exchange is related to the geographic distance separating populations and not strong barriers to dispersal. In addition, both the geographic extent and relative suitability of habitats are predicted to have been reduced at the LGM in comparison to the present (Fig. 5B). The latter result parallels the demographic analyses, which suggest that populations may have increased in size in response to geographic expansion of suitable habitats across Taiwan.

## Discussion

### Evolutionary history

While we found comparatively high levels of genetic variation within populations of Steere's Liocichla, these results demonstrate a lack of genetic differentiation among populations. This is in contrast to the deep genetic divisions reported for other montane species in Taiwan. The haplotype network showed no obvious structure with respect to geography. The AMOVA indicated no genetic differentiation in either mtDNA or microsatellites between north and south groups. However, significant amounts of differentiation were partitioned among populations, implying that populations are not entirely panmictic and that dispersal among populations is likely constrained. This was true of both mtDNA and microsatellites, though the percent of variation among populations was higher for mtDNA. This is expected because mtDNA has, on average, a four times more rapid time to coalescence than nuclear loci [47,48]. Mantel tests were significant when least-cost distance was compared with genetic distance. This suggests that mountains, while not strict barriers to gene flow, do channel gene flow through a certain altitudinal range.

A lack of genetic structure has been found in other mountain species in Taiwan (e.g. White-bellied Rat *Niviventer culturatus*, [49] and Taiwan Alpine Skink *Sphenomorphus taiwanensis*, [50]). This might result from a recent colonization of Taiwan or historical gene flow between populations. We favor a scenario of historical gene flow for Steere's Liocichla, because it is an endemic species that's purported closest relative in Mainland China (*L. omeiensis*; [51]) was estimated to have diverged about 5 million years ago [52]). This suggests that either Steere's Liocichla has existed on Taiwan for a long time or that it has recently gone extinct elsewhere. The idea that populations of Steere's Liocichla have been historically well-connected by gene flow seems plausible given the dispersal potential of volant birds, and the geographic continuity of climatically suitable habitats over time (Fig. 5).

We also find evidence for a recent demographic expansion in Steere's Liocichla. For example, Fu's F_S_, which is particularly sensitive to departures from population equilibrium [23], was negative for most populations and significantly negative when all samples were combined. The distinctly unimodal mismatch distribution did not differ significantly from a model of sudden expansion. The estimated time of this expansion was consistent with an expansion centered around the time of the Last Glacial Maximum. However, it should be noted that there is a large confidence interval associated with this estimate as well as assumptions involving mutation rate and generation time. Ecological niche modeling indicated that there was less suitable habitat for Steere's Liocichla during the LGM. However, marginal habitat seems to have been distributed across the island. This suggests that Steere's Liocichla occupied much of its current range during the LGM, but populations were less dense. Thus, as habitat improved with the warming climate, population densities increased leaving a pattern of recent demographic expansion. This scenario is consistent with the genetic data, which show similar levels of genetic diversity across the island rather than isolated pockets of unusually high diversity that might be interpreted as refugia. Population density increases associated with improving habitat quality following the Last Glacial Maximum might have had an underappreciated contribution to genetic patterns of expansion, especially in cases where dramatic spatial expansion from refugia seems unlikely. It would be interesting to determine whether other montane bird species in Taiwan exhibit similar patterns.

### Conservation implications

Taken separately, the results of some of our analyses could be used as evidence that some populations should be of special management concern for conservation efforts. For example, we found significant differences in microsatellite *F*_*ST *_between the Beidongyanshan population and all other populations. This is surprising because there is no reason to expect restricted gene flow to or from the Beidongyanshan population; it is centrally located, only two km from the Meifeng population, and there is adequate habitat and a continuous population of Steere's Liocichla between Beidongyanshan and Meifeng. However, caution must be taken when interpreting *F*_*ST *_distances associated with highly polymorphic loci. Hedrick [46] has shown that relatively weak bottlenecks can cause statistically significant differences in microsatellite *F*_*ST *_distances. There is evidence that the Beidongyanshan population has recently undergone a population bottleneck and this would explain why its gene frequencies might appear to differ from the other populations.

First, the Beidongyanshan population had the lowest amount of allelic richness. Second, it had a high observed heterozygosity (H_O_) relative to its expected heterozygosity (H_E_), as indicated by the smaller than zero, and nearly significant, *F*_*IS *_value (Table 1). (Note that the AW population has a smaller *F*_*IS *_value, but this likely had a smaller affect on *F*_*ST *_significance because the sample size is much smaller.) A large H_O _relative to H_E _is expected shortly after a population bottleneck because rare alleles are quickly lost, resulting in a decrease in the H_E_. At the same time, H_O _levels are relatively unaffected until a new mutation-drift equilibrium can be reached because H_O _is mostly dictated by common alleles. Such a bottleneck may have been caused by anthropogenic disturbance from the construction of an agricultural research station at Beidongyanshan, from typhoon related disturbance, or from stochastic fluctuations in local demography. A change in *F*_*ST *_caused by a bottleneck is likely ephemeral, existing only until a new mutation-drift equilibrium is reached. Thus, individuals sampled from Beidongyanshan in the future may not show significant *F*_*ST *_differentiation from other populations. We note that in such a situation, *F*_*ST *_may reflect biologically relevant differences between populations, but this differentiation might not actually result from restricted gene flow and might be ephemeral and not of management concern. Clearly, caution should be taken before making conservation recommendations based on the results of *F*_*ST *_alone.

Because it considers information on the length of alleles, *R*_*ST *_is generally thought to perform better than *F*_*ST *_with microsatellite data, especially when there are many alleles. In contrast to *F*_*ST *_results, *R*_*ST *_comparisons did not highlight the Beidongyanshan population as particularly distinct. Instead, the few significant values of *R*_*ST *_do not seem to correlate with geography. For example, there is a significant *R*_*ST *_value between Meifeng and Beidongyanshan, which again are only two km apart, but no significant differences between Meifeng and Moshan or Chilan, which are much farther apart. Overall, significant *R*_*ST *_values seem haphazard with respect to geography. Again, the statistical power associated with highly variable markers, such as microsatellites, is high, so statistically significant differences should not always be taken to mean biologically significant differences [46]. It should also be noted that statically non-random patterns may result from the stochastic nature of population structure [53].

Bayesian analysis of population structure using BAPS found that including the Meifeng population in the pool of remaining populations decreased the fit of a panmictic total, and thus two groups were identified. Again, this is surprising because Meifeng is only two km from Beidongyanshan and there is adequate habitat and a continuous population of Steere's Liocichla between these two populations. However, the spatial analysis in BAPS, which incorporates information about the geographic location of populations, suggested that all populations represented one group. If separating the Meifeng population in the original BAPS analysis was a borderline case, it is understandable that the spatial model suggests only a single population (J. Corander pers. comm.). This was probably caused by an excess of private alleles in the Meifeng population. Possibly as the result of sampling error, Meifeng had an inordinate number of private alleles and all sampled Meifeng individuals had at least one private allele. This would explain why the admixture analysis was able to accurately assign Meifeng individuals to population. Some authors have suggested private alleles are important for conservation. For example, Funk et al. [54] defined subspecies for conservation and included the criteria that they contain "unique alleles or haplotypes." We question the practicality of this particular criterion when deciding conservation priority or making taxonomic decisions because with highly polymorphic loci, most, if not all, populations are likely to contain at least some unique alleles. In our study, all 13 populations we sampled had unique alleles. Overall, some of our analyses might seem to indicate biologically relevant differentiation of a population. However, different analyses disagree about which populations are distinct and no analysis strongly supported the distinctiveness of any population. Therefore, based on these data, none of our sampled populations should have higher conservation priority than any other.

## Conclusions

We did not find the deep genetic divisions reported in other montane species in Taiwan. Instead, populations of Steere's Liocichla have been historically connected by gene flow, though inter-population migration appears largely confined to middle elevations. We found evidence of a demographic expansion that can be dated to the Last Glacial Maximum. Genetic diversity was high across the geographic range, indicating expansion was range-wide, rather than expansion from isolated refugia. Ecological niche modeling corroborated genetic data and suggested that habitat for Steere's Liocichla may have been of poorer quality during the Last Glacial Maximum. Both sources of data are consistent with a single historical scenario--Steere's Liocichla was at lower density during the Last Glacial Maximum and has subsequently expanded in population density. We suggest that such a range-wide density expansion might be an overlooked cause for the genetic patterns of demographic expansion that are regularly reported. We find significant differences among some populations in *F*_*ST *_indices and an admixture analysis. Though both of these results are often used to suggest conservation action, we affirm that statistically significant results are not necessarily biologically meaningful.

## Authors' contributions

BM performed laboratory work, carried out analyses, and drafted the manuscript. HM conceived of the study, collected samples, and participated in the design and coordination of the study. YP collected samples, performed laboratory work, and carried out analyses. KK performed ecological niche modeling analyses and helped draft the manuscript. CY collected samples and participated in the coordination of the study. HY participated in the design and coordination of the study. All authors read and approved the final manuscript.
