# Recent range-wide demographic expansion in a Taiwan endemic montane bird, Steere's Liocichla (Liocichla steerii)

**DOI:** 10.1186/1471-2148-10-153

**Published:** 2010-05-21

**Authors:** Bailey D McKay, Herman L Mays, Yi-Wen Peng, Kenneth H Kozak, Cheng-Te Yao, Hsiao-Wei Yuan, Pei-Fen Lee, Fu-Hsiung Hsu

**Affiliations:** 1 Bell Museum of Natural History, and Department of Ecology, Evolution and Behavior, University of Minnesota, St. Paul, MN 55108, USA; 2 Geier Collections and Research Building, Cincinnati Museum Center, Cincinnati, OH 45203, USA; 3 School of Forestry and Resource Conservation, National Taiwan University, Taipei, Taiwan; 4 Bell Museum of Natural History, and Department of Fisheries, Wildlife & Conservation Biology, University of Minnesota, St. Paul, MN 55108, USA; 5 Endemic Species Research Institute, Council of Agriculture, Jiji, Taiwan; 6 Department of Life Sciences, National Cheng Kung University, Tainan, Taiwan; 7 Institute of Ecology and Evolutionary Biology, National Taiwan University, Taipei, Taiwan; 8 Department of Biological Resources, National Chiayi University,Chiayi, Taiwan

## Correction

After the publication of this work [1], we became aware of three minor errors, none of which have anything to do with the content of our paper. As a result, we have now added an acknowledgment for NSC funding to Hsiao-Wei Yuan. We have also added Hsiao-Wei Yuan as a co-corresponding author. Finally, we have added Pei- Pei-Fen Lee and Fu-Hsiung Hsu to the author list. They provided occurrence data for the Ecological Niche Modeling and felt that they should be co-authors of the study. We apologize for any inconvenience this has caused.
